# Information heterogeneity between progress notes by physicians and nurses for inpatients with digestive system diseases

**DOI:** 10.1038/s41598-024-56324-7

**Published:** 2024-04-01

**Authors:** Yukinori Mashima, Masatoshi Tanigawa, Hideto Yokoi

**Affiliations:** 1 Clinical Research Support Center, Kagawa University Hospital, 1750-1 Ikenobe, Miki-cho, Kita-gun, Kagawa, 761-0793 Japan; 2 Department of Medical Informatics, Faculty of Medicine, Kagawa University, Kagawa, Japan

**Keywords:** Gastroenterology, Data mining, Data processing, Gastrointestinal diseases, Digestive signs and symptoms, Computer science

## Abstract

This study focused on the heterogeneity in progress notes written by physicians or nurses. A total of 806 days of progress notes written by physicians or nurses from 83 randomly selected patients hospitalized in the Gastroenterology Department at Kagawa University Hospital from January to December 2021 were analyzed. We extracted symptoms as the International Classification of Diseases (ICD) Chapter 18 (R00–R99, hereinafter R codes) from each progress note using MedNER-J natural language processing software and counted the days one or more symptoms were extracted to calculate the extraction rate. The R-code extraction rate was significantly higher from progress notes by nurses than by physicians (physicians 68.5% vs. nurses 75.2%; *p* = 0.00112), regardless of specialty. By contrast, the R-code subcategory R10–R19 for digestive system symptoms (44.2 vs. 37.5%, respectively; *p* = 0.00299) and many chapters of ICD codes for disease names, as represented by Chapter 11 K00–K93 (68.4 vs. 30.9%, respectively; *p* < 0.001), were frequently extracted from the progress notes by physicians, reflecting their specialty. We believe that understanding the information heterogeneity of medical documents, which can be the basis of medical artificial intelligence, is crucial, and this study is a pioneering step in that direction.

## Introduction

With the increasingly widespread use of electronic medical records (EMRs), a vast number of medical records are being generated daily and stored electronically. The increase in the amount of accumulated data has brought about investigations for various secondary uses of data^1^, which may be structured or unstructured. Compared with analyses of structured data, analyses of unstructured data, such as narrative text, have been reported to allow for the more accurate extraction of symptoms expressed by patients^2^; therefore, their use has been recommended^3^. However, analyzing narrative text manually not only is labor-intensive, but also can lead to variable results; therefore, natural language processing (NLP) using a computer is indispensable^4^. NLP enables an immense amount of narrative text to be analyzed in a timely and low-cost manner, in addition to the extraction of invariable information^1^. As the use of NLP in medical care progresses, there are high expectations for the realization of quality improvement in medical care and more efficient clinical research^5^. This is also the case in all digestive system fields, including gastrointestinal disease, liver disease, and biliary and pancreatic disease, which would benefit from easier access to clinical information^1^; however, collaboration between NLP and medical experts who can provide domain knowledge is essential to make this practical^6^.

Extracting information such as disease names, symptom names, and adverse events from narrative text in medical documents is one of the goals of NLP research^7,8^, and it is similar in the Japanese domain^9–13^. In recent years, these information extraction techniques have been applied to the development of various predictive models using machine learning, but the risk of misusing the outputs of the system has been pointed out in the process^14–16^. In particular, Hovy et al.^17^ warned that the characteristics of the data to be analyzed are one of the causes of bias and lack of fairness that occur with the use of NLP systems. To promote the correct utilization of NLP in medical care, it is therefore essential to gain a better understanding of the characteristics of various medical documents stored in EMRs from the perspective of NLP.

Various types of medical documents written using narrative text are included in EMRs, such as admission notes, progress notes, and discharge summaries^18,19^; this is no different in Japanese EMRs^20,21^. In particular, progress notes are frequently written by physicians (hereinafter referred to as MD notes) and nurses (hereinafter RN notes)^19^, and are considered to be the most suitable target of analysis for capturing the constantly changing conditions of patients. The biggest difference between MD and RN notes is linked to the different roles that the respective documenters fulfill in medical care. For example, a progress note for the same patient written on the same day by a physician and a nurse will differ because they focus on different things; accordingly, there might be heterogeneity in the information noted. In particular, nurses are specialists at noticing and caring for patients’ symptoms^22,23^, so their notes may contain a substantial amount of symptom-related information. However, few studies have quantitatively examined the differences between MD and RN notes.

Therefore, the present study utilized the NLP software developed by the Nara Institute of Science and Technology, MedNER-J^13,24^. MedNER-J can analyze and extract information from Japanese narrative medical documents and codes complying with the International Statistical Classification of Diseases and Related Health Problems, 10th Revision (hereinafter ICD codes)^25^. In the present study, with the aim of gaining a better understanding of the characteristics of both MD and RN notes, we conducted a quantitative comparison based on extraction rates. In particular, we focused on ICD Chapter 18 (R00–R99, hereinafter R codes), which refer to the names of symptoms, as a possible way to clarify the differences between MD and RN notes and those with occupational backgrounds.

## Methods

### Study population

**Figure 1:**
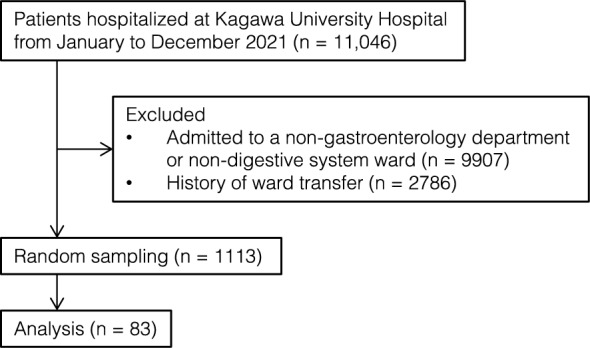
Study population. To unify the organ specialty of the physicians and nurses writing progress notes, we randomly selected 83 patients from among those hospitalized in the Gastroenterology Department and digestive system ward.

Among the 11,046 patients who were hospitalized at Kagawa University Hospital (KUH) from January to December 2021, those who were admitted to the Gastroenterology Department as their main department and to the digestive system ward were targeted for analysis (Fig. 1). KUH is the only national university hospital in Kagawa Prefecture, which is located in the Shikoku region of Japan and has a population of approximately 1 million. To unify the organ specialty of physicians and nurses writing progress notes, we excluded patients who had been admitted to a non-gastroenterology department or a non-digestive system ward (n = 9907) or who had a history of ward transfer (n = 2786). As a result, a total of 1113 eligible patients were identified and randomly enrolled until the patients’ cumulative total hospital stay exceeded 800 days, based on the sample size calculation described below (see the Statistical Analysis section). Finally, a total of 83 patients were enrolled in this study. The total number of hospital days for the 83 patients was 806 days. As background information, we investigated these patients’ age, sex, name of the illness they had been hospitalized for, and duration of hospital stay.

This study was approved by the Research Ethics Committee of Kagawa University Faculty of Medicine through an ethical review (receipt No. 2022-007) and was performed in accordance with the Declaration of Helsinki and the Ethical Guidelines for Medical and Biological Research Involving Human Subjects in Japan. Informed consent was obtained from all patients and/or their legal guardians through an opt-out approach prior to conducting the study.

### Data set preparation

Progress notes for 806 days linked to the 83 patients written by physicians or nurses were collected from the EMR system (HOPE EGMAIN-GX; Fujitsu Ltd., Tokyo, Japan) at KUH and used as an analytical data set. To distinguish the date of each collected progress note, the recording dates for each document were also obtained at the same time (index date). Information about the number of documents, the number of days for which the document existed, the number of documents per hospitalization, and the number of characters per document was also collected. In addition, for all the documents included in the analytical data set, the physicians and nurses who wrote them were identified; the top 10 physicians and nurses who wrote the most documents were also identified and the total number of documents written by these top 10 members was calculated. Furthermore, part of the analytical data set, 106 of 806 days, was used as a validation data set to evaluate the performance of MedNER-J (described below in the Validation of MedNER-J section). The validation data set was created by repeating random inclusion for each patient unit until the total number of hospital days exceeded 10% of the analytical data set^26^. The validation data set consisted of 10 patients.

### Experimental environment

In this study, MedNER-J was operated with the default settings (using a model named BERT, and post-processing using a Japanese disease/symptom name dictionary named J-MeDic^27^) in an environment constructed with the Ubuntu 20.04.3 LTS operating system, the Python 3.8.10 programming language, the MeCab 0.996 Japanese morphological analyzer^28^, and the IPAdic 2.7.0 MeCab dictionary^29^ to extract the ICD codes. MedNER-J extracts the name of the illness from the narrative text of Japanese medical documents by applying conditional random fields to bidirectional encoder representations from transformer embeddings^30^. It also supports the processing of negation and can determine factuality (i.e., distinguish between positive and negative findings). MedNER-J can also assign an ICD code to the extracted illness name by post-processing using J-MeDic. This software is available for public use under the BSD 2-Clause “Simplified” License, and the source code is publicly available on GitHub (https://github.com/sociocom/MedNER-J).

### Validation of MedNER-J

A validation test was performed to evaluate whether the performance of MedNER-J was sufficient for this study. To this end, a board-certified gastroenterologist of the Japanese Society of Gastroenterology (Y.M.) manually reviewed the validation data set to define the gold standard (GS) and determined the applicability of the R codes in each progress note. For each patient, each day (every index date) with one or more expressions that corresponded to an R code was classified as “GS-positive”, and the absence thereof as “GS-negative”. Processing of the validation data set using MedNER-J was performed in parallel. Similarly, for each patient, each day (every index date) with one or more extractions that corresponded to an R code was classified as “MedNER-J-positive,” and the absence thereof as “MedNER-J-negative.” Cohen’s kappa coefficient^31^ with the 95% confidence interval [CI] was calculated as an index of concordance between the GS and MedNER-J for each MD and RN note, and the R-code extraction performance of MedNER-J was evaluated according to the criteria of Landis^32^.

### Comparison between MD and RN notes

**Figure 2:**
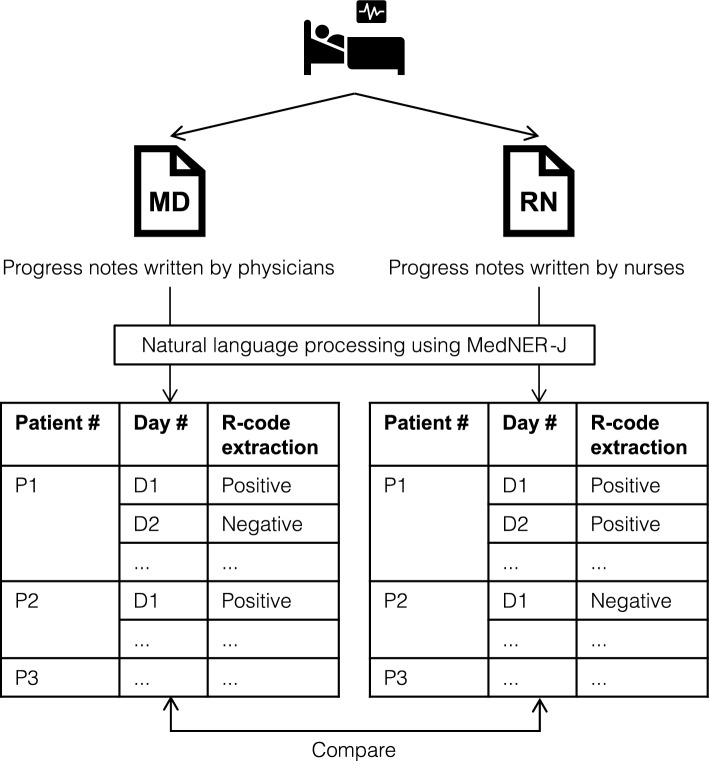
Conceptual diagram. MD and RN notes for the same patients written on the same days were analyzed using MedNER-J, and then compared in terms of R-code extraction rates. Abbreviations: MD notes, progress notes written by physicians; RN notes, progress notes written by nurses; R codes, Chapter 18 R00–R99 (Symptoms, signs and abnormal clinical and laboratory findings, not elsewhere classified) of the International Classification of Diseases.

After processing for the analytical data set using MedNER-J was performed, as a primary evaluation, MD and RN notes were compared using the R-code extraction rates as an indicator (Fig. 2). Notes were classified as “Positive” if there was even one extraction of an R code per patient per day (every index date), and “Negative” otherwise. In the MD and RN notes, the number of “Positive” days was divided by 806 to calculate the R-code extraction rate with the 95% CI, i.e., the percentage of days in which an R code was extracted.

As a secondary evaluation, we compared the ICD code extraction rate from MD and RN notes by R-code subcategories (i.e., by symptom name details), and by chapter-level categories of the ICD codes other than Chapter 18 (R codes), which mainly consists of disease names. As with the primary evaluation, the number of “Positive” days was divided by 806 to calculate the extraction rate (%) with the 95% CI for each ICD code. Furthermore, daily changes in the number of R-code extractions from MD and RN notes were compared per patient, and some patient cases were assessed based on the clinical course.

### Statistical analysis

Based on estimations obtained using G*Power 3.1.9.6^33^, a sample of 796 days was needed to provide 80% power to detect differences in the R-code extraction rate between MD and RN notes, with a two-sided type I error of 0.05, assuming that discordant pairs in the primary evaluation (i.e., “Positive” MD notes against “Negative” RN notes, or “Negative” MD notes against “Positive” RN notes) would occur in one fourth of the total at a ratio of 1.5. In addition, McNemar’s test was performed using R 4.1.2^34^ for hypothesis testing regarding the difference in the proportions in the primary and secondary evaluations. For all analyses, a two-sided *p* value < 0.05 was considered statistically significant.

## Results

### Background characteristics

Table 1 shows the clinical backgrounds of the 83 patients analyzed in the present study (43.4% female; median age, 74 years). Regarding the name of the main illness for hospitalization, gastrointestinal disease was the most common (45.8%), followed by biliary disease (22.9%) and liver disease (19.3%). The median duration of hospital stay per patient was 8 days. Table 2 shows the analytical data set, which consisted of 1415 MD and 3456 RN notes. Of the 806 days for which we attempted to collect progress notes, MD notes were present in 92.4% and RN notes in 99.9%. There was a median of 12 MD and 29 RN notes per hospitalization, respectively. Each MD and RN note had a median of 409 and 122 characters, respectively. The MD notes were written by a total of 74 physicians, while the RN notes were written by a total of 68 nurses. Of these, documents written by the top 10 members in terms of the number of documents written made up 69.0 and 43.9% of MD and RN notes, respectively.

**Table 1:** Clinical backgrounds of the patients.

Total number of patients	83
Age, years^†^	74 [68–80]
Female^‡^	36 (43.4%)
Disease^‡^	
Gastrointestinal disease	38 (45.8%)
Biliary disease	19 (22.9%)
Liver disease	16 (19.3%)
Pancreatic disease	5 (6.0%)
Other diseases	5 (6.0%)
Total number of hospital days	806
Hospital duration per patient, days^†^	8 [4.5–11.5]

^†^Data are expressed as median [interquartile range].

^‡^Data are expressed as number (percentage in 83 patients).

**Table 2:** Characteristics of the data set.

	MD notes	RN notes
Total number of documents	1415	3456
Number of days the document existed^†^	745 (92.4%)	805 (99.9%)
Number of documents per hospitalization^‡^	12 [7–19]	29 [20–51.5]
Number of characters per document^‡^	409 [214–682.5]	122 [63–195]
Number of physicians or nurses who wrote the document	74	68
Number of documents written by the top 10 physicians or nurses who wrote the most documents^§^	977 (69.0%)	1517 (43.9%)

^†^Data are number of days (percentage in 806 days).

^‡^Data are expressed as median [interquartile range].

^§^Data are number of documents (percentage in total documents).

MD notes, progress notes written by physicians; RN notes, progress notes written by nurses.

### Performance of MedNER-J

In the validation data set, the MD and RN notes were defined as “GS-positive” in 80 (74.5%) and 97 (91.5%) of the 106 days, respectively. By contrast, the MD and RN notes were defined as “MedNER-J-positive” in 81 (76.4%) and 92 days (86.8%), respectively. As for the concordance between GS and MedNER-J, Cohen’s kappa coefficients for the MD and RN notes were 0.61 (95% CI 0.44–0.79) and 0.66 (95% CI 0.43–0.89), respectively, both showing substantial concordance according to the criteria of Landis.

### Comparison by R codes and other ICD codes

**Figure 3:**
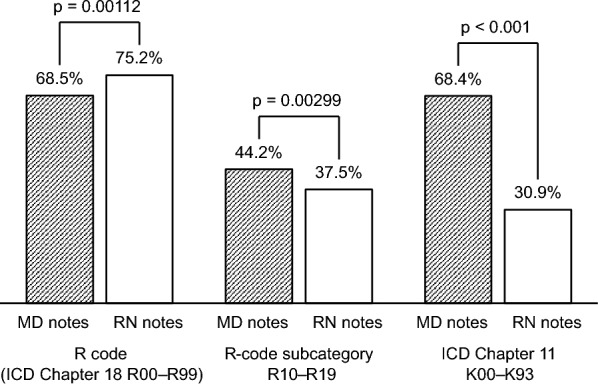
Comparison of extraction rates in three categories of ICD codes. MD notes and RN notes are compared and shown in terms of each code extraction rate. R code, i.e., ICD Chapter 18 R00–R99, consists of symptom names, and R-code subcategory R10–R19 consists of names of digestive system symptoms. Meanwhile, ICD Chapter 11 K00–K93 consists of disease names of the digestive system. Abbreviations: MD notes, progress notes written by physicians; RN notes, progress notes written by nurses; ICD, International Classification of Diseases.

Tables 3 and 4 show the extraction rates for each code from the MD and RN notes, calculated by category. Three notable categories of these results are shown in Fig. 3. The R-code extraction rates from the MD and RN notes using MedNER-J were 68.5% (552 days) and 75.2% (606 days), respectively, of the 806 days in the analytical data set. For R-code extraction, discordant pairs occurred in 33.0% (266 days). The R-code extraction rate from RN notes was significantly higher (*p* = 0.00112). When comparing by R-code subcategories, the extraction rates for R10–R19, which are names of digestive system symptoms, from the MD and RN notes were 44.2 and 37.5%, respectively. The extraction rate for R10–R19 from MD notes was significantly higher than that from RN notes (*p* = 0.00299). Conversely, the extraction rate from RN notes was significantly higher than that for many other R-code subcategories, such as R40–R46 (MD notes 8.4% vs. RN notes 19.4%, p < 0.001) and R50–R69 (44.0 vs. 59.4%, respectively; *p* < 0.001). When comparing by chapter-level categories of ICD codes, the extraction rates for Chapter 11 K00–K93, which are disease names of the digestive system, were 68.4% from MD notes and 30.9% from RN notes (*p* < 0.001); i.e., the extraction rate from MD notes was significantly higher. Excluding Chapter 5 F00–F99 and Chapter 19 S00–T98, the extraction rate from MD notes was significantly higher in most of the other chapter-level categories of the ICD codes.

**Table 3:** Comparison between MD and RN notes by R code and subcategory.

		Extraction rate		*p* value
		MD notes	RN notes	
R code (Chapter 18 of the ICD codes)				
R00–R99	Symptoms, signs and abnormal clinical and laboratory findings, not elsewhere classified	68.5% (65.2–71.7)	75.2% (72.1–78.1)	0.00112
R-code subcategories				
R00–R09	Symptoms and signs involving the circulatory and respiratory systems	10.7% (8.6–13.0)	11.2% (9.1–13.5)	0.78
R10–R19	Symptoms and signs involving the digestive system and abdomen	44.2% (40.7–47.7)	37.5% (34.1–40.9)	0.00299
R20–R23	Symptoms and signs involving the skin and subcutaneous tissue	9.7% (7.7–11.9)	10.2% (8.2–12.5)	0.80
R25–R29	Symptoms and signs involving the nervous and musculoskeletal systems	1.5% (0.8–2.6)	6.6% (5.0–8.5)	< 0.001
R30–R39	Symptoms and signs involving the urinary system	1.0% (0.4–1.9)	2.7% (1.7–4.1)	0.00936
R40–R46	Symptoms and signs involving cognition, perception, emotional state and behaviour	8.4% (6.6–10.6)	19.4% (16.7–22.3)	< 0.001
R47–R49	Symptoms and signs involving speech and voice	4.0% (2.7–5.6)	2.9% (1.8–4.3)	0.0225
R50–R69	General symptoms and signs	44.0% (40.6–47.6)	59.4% (55.9–62.8)	< 0.001
R70–R79	Abnormal findings on examination of blood, without diagnosis	1.9% (1.0–3.1)	0.1% (0.0–0.7)	< 0.001
R80–R82	Abnormal findings on examination of urine, without diagnosis	0.5% (0.1–1.3)	0.2% (0.0–0.9)	0.69
R83–R89	Abnormal findings on examination of other body fluids, substances and tissues, without diagnosis	0.0% (0.0–0.5)	0.0% (0.0–0.5)	n/a
R90–R94	Abnormal findings on diagnostic imaging and in function studies, without diagnosis	9.6% (7.6–11.8)	0.0% (0.0–0.5)	< 0.001
R95–R99	Ill-defined and unknown causes of mortality	0.0% (0.0–0.5)	0.1% (0.0–0.7)	1.0

Data are expressed as percentage in 806 days and 95% confidence intervals.

MD notes, progress notes written by physicians; RN notes, progress notes written by nurses; ICD, International Classification of Diseases; n/a, not applicable.

**Table 4:** Comparison between MD and RN notes by ICD codes other than R codes.

			Extraction rate		*p* value
			MD notes	RN notes	
Chapter 1	A00–B99	Certain infectious and parasitic diseases	16.6% (14.1–19.4)	7.6% (5.8–9.6)	< 0.001
Chapter 2	C00–D48	Neoplasms	65.6% (62.2–68.9)	11.0% (9.0–13.4)	< 0.001
Chapter 3	D50–D89	Diseases of the blood and blood-forming organs and certain disorders involving the immune mechanism	7.4% (5.7–9.5)	1.9% (1.0–3.1)	< 0.001
Chapter 4	E00–E90	Endocrine, nutritional and metabolic diseases	41.7% (38.3–45.2)	2.9% (1.8–4.3)	< 0.001
Chapter 5	F00–F99	Mental and behavioural disorders	5.3% (3.9–7.1)	12.2% (10.0–14.6)	< 0.001
Chapter 6	G00–G99	Diseases of the nervous system	13.0% (10.8–15.5)	6.9% (5.3–8.9)	< 0.001
Chapter 7	H00–H59	Diseases of the eye and adnexa	4.8% (3.5–6.6)	1.7% (1.0–2.9)	< 0.001
Chapter 8	H60–H95	Diseases of the ear and mastoid process	5.0% (3.6–6.7)	2.4% (1.4–3.7)	0.00309
Chapter 9	I00–I99	Diseases of the circulatory system	48.4% (44.9–51.9)	13.8% (11.5–16.3)	< 0.001
Chapter 10	J00–J99	Diseases of the respiratory system	21.1% (18.3–24.1)	6.7% (5.1–8.7)	< 0.001
Chapter 11	K00–K93	Diseases of the digestive system	68.4% (65.0–71.6)	30.9% (27.7–34.2)	< 0.001
Chapter 12	L00–L99	Diseases of the skin and subcutaneous tissue	13.9% (11.6–16.5)	6.7% (5.1–8.7)	< 0.001
Chapter 13	M00–M99	Diseases of the musculoskeletal system and connective tissue	14.1% (11.8–16.7)	7.1% (5.4–9.1)	< 0.001
Chapter 14	N00–N99	Diseases of the genitourinary system	17.7% (15.2–20.6)	1.2% (0.6–2.3)	< 0.001
Chapter 15	O00–O99	Pregnancy, childbirth and the puerperium	1.0% (0.4–1.9)	0.0% (0.0–0.5)	0.00781
Chapter 16	P00–P96	Certain conditions originating in the perinatal period	0.0% (0.0–0.5)	0.0% (0.0–0.5)	n/a
Chapter 17	Q00–Q99	Congenital malformations, deformations and chromosomal abnormalities	2.1% (1.2–3.4)	0.2% (0.0–0.9)	< 0.001
Chapter 19	S00–T98	Injury, poisoning and certain other consequences of external causes	14.1% (11.8–16.7)	23.2% (20.3–26.3)	< 0.001
Chapter 20	V01–Y98	External causes of morbidity and mortality	0.2% (0.0–0.9)	0.0% (0.0–0.5)	0.50
Chapter 21	Z00–Z99	Factors influencing health status and contact with health services	1.1% (0.5–2.1)	0.0% (0.0–0.5)	0.00391
Chapter 22	U00–U99	Codes for special purposes	0.0% (0.0–0.5)	0.0% (0.0–0.5)	n/a

Data are expressed as percentage in 806 days and 95% confidence intervals.

MD notes, progress notes written by physicians; RN notes, progress notes written by nurses; ICD, International Classification of Diseases; R codes, Chapter 18 of the ICD codes; n/a, not applicable.

### Case presentation

#### Case 1

**Figure 4:**
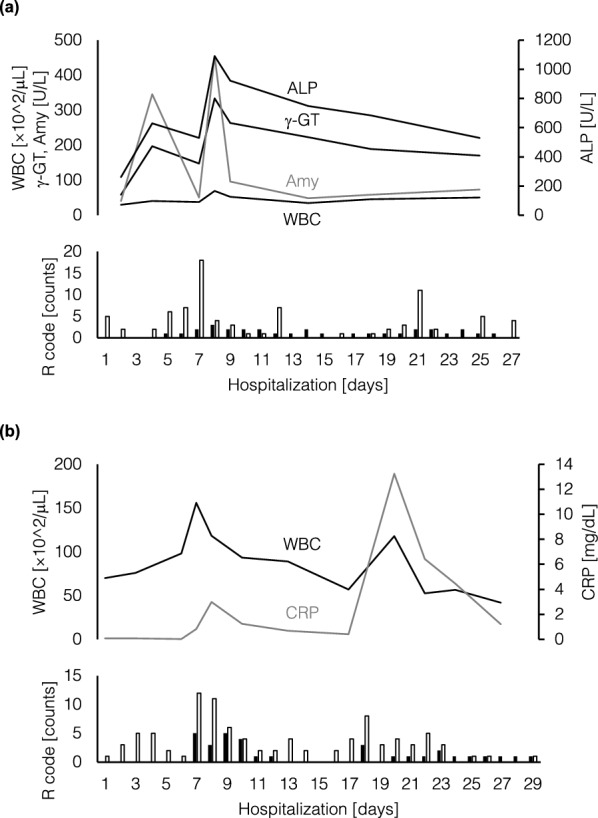
Case presentation. The lines indicate the laboratory test values, and the bars indicate the number of R-code extractions from each progress note (Black, MD notes; White, RN notes). Some lines are colored gray for readability. (**a**) The patient was a woman in her 70 s diagnosed with an infected pancreatic cyst. (**b**) The patient was a woman in her 70 s diagnosed with hepatocellular carcinoma. Abbreviations: R codes, Chapter 18 R00–R99 (Symptoms, signs and abnormal clinical and laboratory findings, not elsewhere classified) of the International Classification of Diseases; MD notes, progress notes written by physicians; RN notes, progress notes written by nurses; ALP, alkaline phosphatase; γ-GT, gamma glutamyl transpeptidase; Amy, amylase; WBC, white blood cell count; CRP, C-reactive protein.

Case 1 was a woman in her 70 s diagnosed with an infected pancreatic cyst (Fig. 4a). Previously, she had been hospitalized for more than 1 month and received conservative treatment. Her condition was improved by fasting, but recurred once her normal diet was resumed. Various causes were investigated by the previous physician, but no clear cause was identified; therefore, she was referred to KUH. Her symptoms had subsided on Day 2 at KUH, so the reintroduction of her diet was attempted, but fever was observed again on Day 5. She fasted again and was followed up, but her fever and laboratory findings suddenly exacerbated on Day 8. On the same day, emergency abdominal echography showed common bile duct stones. Emergency endoscopic biliary and pancreatic stenting were performed, and she was cured without recurrence even after the resumption of her diet. Endoscopic lithotripsy was performed on Day 24, and she was discharged on Day 27. The number of R-code extractions from RN notes exceeded that from MD notes during most of the days during the patient’s hospitalization. The number of R-code extractions from RN notes increased dramatically on Days 7 and 21. In particular, the R codes extracted from RN notes on Day 7 were examined individually, revealing that codes that would allow for prediction of exacerbation on Day 8, such as R10 (Abdominal and pelvic pain) and R50 (Fever of other and unknown origin), were extracted multiple times.

#### Case 2

Case 2 was a woman in her 70 s, diagnosed with hepatocellular carcinoma with underlying hepatitis B virus-related cirrhosis, who was admitted to KUH for treatment (Fig. 4b). As initially planned, transcatheter arterial chemoembolization (TACE) was performed on Day 2. She was following a favorable postoperative course for several days, but bloody stool and abdominal pain were observed on Day 7. Radiofrequency ablation (RFA) was originally planned for Day 8, but was suddenly postponed. As a result of the search for the cause of the bloody stool, an unexpected complication, the patient was diagnosed with acute hemorrhagic colitis associated with prophylactic antibiotic therapy for TACE. The bloody stool did not progress to anemia, and her condition improved by discontinuing the suspected cause to observe her course. On Day 16, the postponed RFA was performed, and in preparation, another antibiotic from what was believed to have caused the bloody stool was administered for prophylaxis. Her bloody stool did not reappear after RFA, but she complained of abdominal pain again on Day 18. As the abdominal pain was an expected symptom after the procedure, she received follow-up with symptomatic therapy alone. Her abdominal pain subsequently disappeared, and she was discharged on Day 29. Regarding the laboratory test values, hemoglobin remained within the normal range (data not shown in the figure), and indicators of inflammation such as white blood cell count and C-reactive protein remained within the permissible range after RFA. The number of R-code extractions from the RN notes exceeded that from the MD notes during most of the days of the patient’s hospitalization, similar to Case 1. Both increased several days after each procedure, but a particularly noticeable peak was observed from Days 7 to 10. An individual examination of the extracted R codes showed that R58 (Haemorrhage, not elsewhere classified) was extracted with the appearance of bloody stool in both progress notes. Moreover, codes such as R42 (Dizziness and giddiness), representing symptoms suspected to have occurred secondary to a hemorrhagic event, were extracted from only the RN notes.

## Discussion

To our knowledge, this is the first study to show information heterogeneity between MD and RN notes through a comparison of R-code extraction rates, which were calculated based on symptom names from each note written for the same patient on the same day using MedNER-J. To carry out this study, MedNER-J showed sufficient performance when compared with a chart review by a human expert for both MD and RN notes. The content of medical documents is affected by not only the patient or disease condition, but also various other factors, including the documenter’s experience^35^, so the presence of information heterogeneity between MD and RN notes is understandable. The heterogeneity observed in this study is a bias that should be recognized by NLP system developers. At the same time, the findings suggest the importance of interpreting the output with an understanding of the characteristics of each medical document by NLP system users. In particular, many systems that utilize artificial intelligence algorithms are more affected by their training data^36^; therefore, medical documents that are used as training data should be treated with special measures to prevent erroneous decision-making.

The analytical data set used in the present study included several days when there were no MD notes, whereas RN notes existed for almost all days; this might have been affected by the work schedules of the physicians and nurses. In other words, attending physicians in Japanese hospitals generally work overtime on weekends and holidays only when necessary to respond to emergencies^37^, whereas nurses take shifts and provide care without any breaks in between and throughout weekends and holidays^38^. These differences in the work schedules of physicians and nurses may have been reflected in the differences in the number of days when progress notes were available out of the total number of days during patient hospitalizations. Another difference was that, while there were fewer documents themselves in terms of MD notes per hospitalization compared with RN notes, there were more characters per document. Whereas physicians summarized the daily clinical course of a patient in one document, nurses seemed to be recording details across several documents.

Although a significantly higher R-code extraction rate was observed for RN notes, R-code subcategory R10–R19 was extracted significantly more from MD notes. In comparisons by chapter-level categories of ICD codes, MD notes had a significantly higher extraction rate for most categories, except for Chapter 5 F00–F99 and Chapter 19 S00–T98. These results suggest that MD notes are written using symptom and disease names according to the documenter’s specialty (in this study, the digestive system domain), but are mainly written using disease names for fields outside of their specialty. On the other hand, nurses generally used disease names infrequently, but used symptoms for various fields without being biased toward a specific domain when writing progress notes. This may be linked to the Japanese Medical Practitioners’ Act, which stipulates that “No person except a medical practitioner may engage in medical practice” because making diagnoses is generally considered an absolute medical practice, and only physicians have permission to diagnose exclusively^39^. The history and availability of nurse practitioners who are allowed to make diagnoses partially are still limited in Japan^40^, and such nurses were not working at KUH, at least not during the study period. In addition, a study comparing the terminology used by physicians and nurses showed that nurses write the patient’s condition specifically using a variety of terms and expressions, thereby aiming for close information-sharing between nurses and realizing better care practice^41^. The occupational backgrounds of nurses may also explain why RN notes had fewer expressions of disease names but numerous expressions of symptom names.

Furthermore, in the two case presentations, the R-code extractions from the two types of documents generally matched clinical events, visually speaking. In particular, R-code extractions from RN notes showed sharp fluctuations. In part, the increase in R-code extractions from RN notes was earlier than the worsening of laboratory test values. Generally, there are many clinical conditions in which the patient’s symptoms change before laboratory values, suggesting that this may be reflected by changes in the number of R-code extractions from RN notes. A classic textbook on abdominal examination^42^, Cope’s Early Diagnosis of the Acute Abdomen, also explained that more diagnoses will be made through the history of a patient’s condition than through various tests, and health-care practitioners must be aware of the earliest symptoms to recognize the early stages of the disease^43^.

The results of this study suggest that accurate extraction of R codes from RN notes is one of the most important factors in considering the development of future clinical decision support systems using narrative documents in EMRs. In other words, the real-time signal detection of changes in the patient’s condition based on R-code extraction from RN notes, and the notification of such changes to physicians, may be the key to an early diagnosis. Previous studies on the development of information extraction algorithms^44^ or prognosis prediction algorithms^45^ have reported the advantages of using nursing records as a data source compared with physician records only. Based on the findings of the present study, we posit that the diverse range of symptom descriptions within nursing records, unbiased toward any specific specialty, could positively contribute to the effectiveness of machine learning. These findings also emphasize the importance of daily documentation in nursing practice, especially in terms of recording patients’ symptoms. In addition, the present findings highlight the importance of information heterogeneity in medical documents, which should be noted when considering the secondary use of medical documents using NLP. The analysis of medical documents by NLP is expected to improve efficiency in the field of clinical research^1^; therefore, clarification is required as to whether the information to be collected is a disease name or a symptom name. In other words, MD notes should be analyzed if the intention is to collect the disease name, whereas RN notes should be analyzed if the intention is to collect the symptom name. Thus, it is necessary to select the applicable subject for the analysis carefully, taking the information heterogeneity of medical documents into account. Furthermore, as adopted in the present study, by extracting essential information from progress notes using NLP in an efficient and structured manner as standardized codes such as R codes or ICD codes, the extraction frequency of each code may be utilized as a quality indicator for medical safety management. For example, if a significant code extraction is found from the progress notes, it will be possible to detect this as a signal of some kind of abnormality and take appropriate clinical action. We expect that the extraction of standardized codes from progress notes will be further explored in the future.

This study has several strengths. To our knowledge, this is the first report to compare quantitatively the amount of information in physician and nursing records from the perspective of NLP. Various reports have attempted to extract information from medical documents^7–13^, but none of these studies have compared quantitatively the amount of information between different documents linked to identical patients. We achieved this comparison by converting various narrative representations into standardized codes. Second, a well-planned research protocol was designed for the present study. The NLP software used in this study was validated based on GS definitions by human experts; we believe that this process improved the reliability of the results in the comparison among documents in the second half of the study. This is also the first report to visualize the increase in symptom names in physician and nursing records with a corresponding increase in the level of medical need. On the other hand, this study also has some limitations. First, it was conducted at a single institution and within a single disease domain. Furthermore, as shown in Table 2, the analytical data set contained progress notes written at a high rate by specific physicians or nurses. Therefore, it is possible that diversity in progress notes by institution, disease domain, or documenter has not been sufficiently considered. Second, this study was an experiment using MedNER-J NLP software. If different NLP software had been used for analysis, it might have changed the code extraction performance, and consequently, the code extraction rate. Third, here, we focused only on inpatient progress notes; outpatient progress notes were out of the scope of the present study. In outpatient care, the frequency and content of RN notes are expected to be quite different. Lastly, this study was conducted in Japan, where the nurse practitioner system is not widespread. Under the nurse practitioner system, specific nurses are responsible for some of the diagnoses, in which case RN notes likely include more disease names than what was found in the present analysis.

## Conclusion

The findings of this study showed quantitative differences in that nurses wrote symptoms more frequently in progress notes than did physicians. By contrast, physicians wrote more disease names in progress notes than did nurses. However, limited to their specialty fields, physicians also wrote symptoms frequently. On the other hand, no biases by organ specialty were found in RN notes. In addition, there were almost no days throughout the patient’s hospitalizations in which RN notes were not written, and RN notes were written multiple times a day. These results suggest that extracting R codes from RN notes allows the extraction of more fine-grained information about patient conditions that might change day-by-day. Therefore, gaining a better understanding of the characteristics of each medical document is necessary for the proper application of NLP in medical care.

## Data Availability

The data sets analyzed in this study are available from the corresponding author on reasonable request, with the participants’ personal information removed.
